# Sepiapterin Improves Vascular Reactivity and Insulin-Stimulated Glucose in Wistar Rats

**DOI:** 10.1155/2018/7363485

**Published:** 2018-09-23

**Authors:** A. C. Keller, L. A. Knaub, R. L. Scalzo, S. E. Hull, A. E. Johnston, L. A. Walker, J. E. B. Reusch

**Affiliations:** ^1^Division of Endocrinology, University of Colorado Anschutz Medical Campus, Aurora, CO, USA; ^2^Department of Medicine, Denver VA Medical Center, Denver, CO, USA; ^3^Center for Women's Health Research, University of Colorado School of Medicine, Aurora, CO, USA; ^4^Division of Cardiology, University of Colorado, Anschutz Medical Campus, Aurora, CO, USA

## Abstract

In the vasculature, sedentary behavior leads to endothelial abnormalities, resulting in elevated cardiovascular disease risk. Endothelial nitric oxide synthase (eNOS) aberrations characterize endothelial dysfunction; eNOS also regulates mitochondrial function. We hypothesized that sepiapterin (a precursor to eNOS cofactor tetrahydrobiopterin (BH_4_)) supplementation would improve endothelium-dependent vascular relaxation in sedentary animals via modulation of NOS function and mitochondrial activity. Sedentary male Wistar rats were fed ad libitum for a total of 10 weeks. Sepiapterin was administered in diet during the final 5 weeks. Intraperitoneal insulin and glucose tolerance tests (IP-ITT/IP-GTT) were conducted at baseline and endpoint. Aorta was assessed for vasoreactivity and mitochondrial respiration. Insulin tolerance, determined by IP-ITT, significantly improved in rats treated with sepiapterin (*p* < 0.05, interaction of time and treatment). Acetylcholine- (ACh-) driven vasodilation was significantly greater in aorta from sepiapterin-treated rats as compared with control (76.4% versus 54.9% of phenylephrine contraction at 20 *μ*M ACh, *p* < 0.05). Sepiapterin treatment resulted in significantly elevated state 3 (9.00 oxygen pmol/sec^∗^mg versus 8.17 oxygen pmol/sec^∗^mg, *p* < 0.05) and 4 (7.28 oxygen pmol/sec^∗^mg versus 5.86 oxygen pmol/sec^∗^mg, *p* < 0.05) aortic mitochondrial respiration with significantly lower respiratory control ratio (*p* < 0.05) during octanoylcarnitine-driven respiration. Vasodilation and insulin sensitivity were improved through targeting NOS via sepiapterin supplementation.

## 1. Introduction

Sedentary behavior, considered a leading cause of death by the Centers for Disease Control and Prevention, is associated with health risks including cardiovascular disease (CVD) risk, sleep problems, decreased quality of life, and endothelial dysfunction in middle-aged adults [1–9]. Clinical, preclinical, and epidemiological data all show strong links between sedentary behavior and endothelial dysfunction, decreased cardiorespiratory fitness, and increased vascular intimal medial thickness [4–7]. One of the primary regulators of endothelial function is endothelial nitric oxide synthase (eNOS), and its activity is impaired in endothelial dysfunction [10–12]. eNOS dysfunction is also a precursor to CVD and predictive of CVD risk [10, 12, 13]. Recent clinical studies demonstrate that sedentary behavior may result in diminished NO availability [14]. Cohort analysis demonstrates that increased exercise across the life span preserves NOS [14]. This background suggests that maintenance of eNOS function in the sedentary state could improve vascular function.

Our laboratory and others have shown that eNOS regulates vascular dilation and it is also upstream of vascular mitochondrial function [15–18]. Experimental data demonstrate the importance of NOS for mitochondrial content, mass, function, quality, and turnover in the vasculature [16, 17]. Our and others' work supports that mitochondria are adversely impacted by chronic disease and nutrient stress [11, 19]. Sedentary behavior likely has adverse effects on both eNOS and mitochondrial function in the vasculature. However, it is unknown whether targeting eNOS activity will improve vascular mitochondrial respiration and function and thereby alleviate endothelial dysfunction correlated with sedentary behavior. Thus, supporting eNOS function is a straightforward strategy for repairing vascular function.

eNOS activation and NO production occur when the enzyme is in a dimer formation, referred to as coupling; this dimer structure is facilitated in part by the cofactor tetrahydrobiopterin (BH_4_) [20]. One mechanism of eNOS disruption occurs when the enzyme becomes uncoupled and generates reactive oxygen species (ROS) instead of NO [20]. eNOS uncoupling occurs in disease states with elevated ROS, such as diabetes [11, 21]. Although supplementation with BH_4_ can be an approach for supporting eNOS activity, BH_4_ is subject to oxidation and degradation in the context of excess ROS [22]. Thus, studies incorporating BH_4_ to support eNOS function have shown mixed results depending upon the balance of oxidants and reducing agents in the environment [23]. To bypass this problem, an experimental strategy to increase BH_4_ in the setting of elevated ROS employs the BH_4_ precursor sepiapterin. Sepiapterin appears to be oxidant-resistant in that it is reported to significantly increase endogenous BH_4_ and NO concentrations and recouple eNOS in endothelial cells, even in the context of ambient ROS [24]. Sepiapterin supplementation in diabetic or obese mice resulted in a significant increase in BH_4_, coupled eNOS, and NO production [4, 24–27], and diabetic mice fed sepiapterin along with L-citrulline showed inhibition of diabetic cardiomyopathy as well as beneficial effects on eNOS dimerization and NO production [28]. In a rat model of left ventricle hypertrophy, sepiapterin intake attenuated this condition while increasing NO [29]. These studies demonstrate that sepiapterin restores BH_4_ concentrations, eNOS function, and NO production, resulting in downstream functional benefits in the heart; however, the effects of this BH_4_ restoration on mitochondrial function and vasoreactivity in sedentary animals are unexamined.

We hypothesized that sepiapterin supplementation would improve endothelium-dependent vascular relaxation in sedentary animals via the augmentation of eNOS function and mitochondrial activity. Sedentary animals consuming sepiapterin showed improvement in vasodilation and modulations in mitochondrial respiration. Further, assessment of the metabolic status of these sedentary animals revealed improved glucose disposal in the presence of insulin in the rats treated with sepiapterin.

## 2. Materials and Methods

Glucose was measured using a FreeStyle Lite® (Abbott) glucometer with accompanying strips, and triglyceride assay reagents were procured from Sigma. Insulin (Humulin R, Lilly) was purchased at a local pharmacy. Insulin ELISA kits were from ALPCO Diagnostics. S-Nitro-N-acetyl-DL-penicillamine (SNAP) and cyclic guanosine monophosphate (cGMP) were procured from Cayman Chemical Company. Sodium chloride, potassium chloride, calcium chloride, magnesium chloride, sodium bicarbonate, potassium phosphate, D-glucose, collagenase, ethylenediaminetetraacetic acid (EDTA), ethylene glycol tetraacetic acid (EGTA), sodium pyrophosphate, sodium orthovanadate, sodium fluoride, okadaic acid, 1% protease inhibitor cocktail, dithiothreitol, magnesium chloride, K-lactobionate, taurine, potassium phosphate, HEPES, digitonin, pyruvate, malic acid, glutamic acid, adenosine diphosphate, succinic acid, oligomycin, carbonyl cyanide 4-(trifluoromethoxy)phenylhydrazone, phenylephrine and ACh, trypsin inhibitor, and cytochrome c were procured from Sigma-Aldrich (MO, USA). Mammalian Protein Extraction Reagent (M-PER) was from Thermo Scientific, and Immobilon-P PVDF membrane was from Millipore. Sepiapterin was procured from Sigma-Aldrich.

### 2.1. Antibodies

Antibodies to adenosine monophosphate kinase (AMPK, rabbit), phosphorylated AMPK (pAMPK, rabbit, Thr172), and sirtuin 3 (SIRT3, rabbit), were purchased from Cell Signaling; eNOS (mouse) was from BD Biosciences; antibody cocktail (Total OXPHOS Blue Native WB Antibody Cocktail) with representative subunits of mitochondrial oxidative phosphorylation (OxPhos) complexes I (subunit NDUFA9), II (subunit SDHA), III (subunit UQCRC2), IV (subunit IV), and V (subunit ATP5A) (mouse) was procured from Abcam; phosphorylated eNOS (peNOS, rabbit, S1177) and peroxisome proliferator-activated receptor gamma (PPAR*γ*) coactivator 1 alpha (PGC-1*α*, rabbit) were purchased from Santa Cruz Biotechnology; and protease inhibitor cocktail and antibody to *β*-actin (mouse) were from Sigma. Secondary antibodies (1 : 10,000, mouse, and 1 : 10,000, rabbit) for Western blot detection were purchased from Li-COR.

### 2.2. Animals

Animal usage and experimental design and procedures were approved by the University of Colorado Denver Institutional Animal Care and Use Committee. Our treatment of animals was in accordance with the guidelines stipulated by the National Institutes of Health. Male Wistar rats were provided by Charles River Laboratories. Animals were kept in the University of Colorado Denver facility in a 12 : 12 light cycle with access to water and food ad libitum.

### 2.3. Experimental Details

Animals (male Wistar rats, 8 weeks old), housed 2 animals per cage, were fed a customized diet containing 13% kcal fat (Envigo [Teklad]) for 10 weeks. Animals were randomized into either control (*n* = 5) or sepiapterin (*n* = 4) groups, and after the first five weeks, the control group continued on the diet and the sepiapterin group was given food containing sepiapterin at 10 mg/kg body weight ad libitum [25, 29]. Food consumption was measured and food replaced twice per week. Blood was taken biweekly for ad libitum glucose and insulin concentrations, and weights were taken weekly. Endpoint parameters were taken at sacrifice, and all animals were euthanized in the morning following ad libitum food consumption.

### 2.4. Insulin and Glucose Intraperitoneal Tolerance Tests

Insulin tolerance testing (ITT) was done at baseline and endpoint of the study, following a 6-hour fast, by injection of 1 U/kg body weight of insulin. Blood glucose concentrations were sampled at baseline and 15, 30, 45, 60, and 120 minutes post injection. Glucose tolerance testing (GTT) followed the same protocol using 1.5 g/kg body weight of glucose, injected intraperitoneally. All animals were included in testing; however, statistics were only performed on *n* = 3 in the sepiapterin group for ITT at the end of the study due to a missing sample at one of the time points.

### 2.5. Vasoreactivity

Aortae were taken from rats at sacrifice (control group *n* = 5, sepiapterin *n* = 4), cleaned of fat and tissue, and measured for vasoreactivity using force tension as previously described [11, 30–32]. Denuding was accomplished by mechanical means; interior tissue was rubbed gently with tweezers. Denuding was considered successful by Student *t*-test comparison of control intact and denuded aorta; this showed that denuded tissue had significantly less response to ACh as compared with intact (*p* = 0.0027, data not shown). Briefly, tissue (2 mm rings) was mounted on a stainless steel hook and a force-displacement transducer (Grass Instruments Co.) while being incubated in a bath at 1.5 g basal tension; baths contained Krebs buffer (119 mM NaCl, 4.7 mM KCl, 2.5 mM CaCl_2_, 1 mM MgCl_2_, 25 mM NaHCO_3_, 1.2 mM KH_2_PO_4_, and 11 mM D-glucose) and continuously bubbled with 95% O_2_ and 5% CO_2_. Aorta constriction was conducted by exposure to 80 mM KCl. A phenylephrine dose response curve was also done with doses ranging from 0.002 *μ*M to 0.7 *μ*M concentrations. To investigate vasodilation, a dose response curve with ACh was performed with a range of 0.05 *μ*M to 20.0 *μ*M concentrations. Aortae were also exposed to 10 *μ*M of a nitric oxide donor (S-nitro-N-acetyl-DL-penicillamine, SNAP) as well as 125 *μ*M cGMP. Data was collected using AcqKnowledge software.

### 2.6. Mitochondrial Respiration

Mitochondrial respiration was measured using Oroboros Oxygraph-2k (O2k; Oroboros Instruments Corp.). The aortae (control group *n* = 5, sepiapterin *n* = 4) was removed and placed in a mitochondrial preservation buffer [BIOPS (10 mM Ca-EGTA, 0.1 mM free calcium, 20 mM imidazole, 20 mM taurine, 50 mM K-MES, 0.5 mM DTT, 6.56 mM MgCl_2_, 5.77 mM ATP, and 15 mM phosphocreatine, pH 7.1)] and kept on ice. The aortic endothelium was removed with a cotton-tipped applicator and then permeabilized by incubation with saponin (40 mg/mL) in BIOPS on ice on a shaker for 30 minutes. It was then washed for 10 minutes on ice on a shaker in mitochondrial respiration buffer [MiR06 (0.5 mM EGTA, 3 mM magnesium chloride, 60 mM K-lactobionate, 20 mM taurine, 10 mM potassium phosphate, 20 mM HEPES, 110 mM sucrose, 1 g/L bovine serum albumin, 280 U/mL catalase, pH 7.1)]. The aorta was blotted dry for weight measurements (8–20 mg) and added to MiR06 that had been prewarmed to 37°C in the chamber of O2k. Oxygen concentration in MiR06 started at approximately 400 mM and was maintained above 250 mM. Substrates and inhibitors were added to assess respiration rates at several states, including background consumption with carbohydrate or lipid only (state 2), oxidative phosphorylation (+ADP, state 3), maximum oxidative phosphorylation (succinate, state 3S), state 4 (+oligomycin), and uncoupled state (+FCCP). In experiment 1 (pyruvate/malate/glutamate-driven), respiration rates were measured with the final concentrations of 5 mM pyruvate + 2 mM malate + 10 mM glutamate, 2 mM adenosine diphosphate (ADP), 6 mM succinate, 4 mg/mL oligomycin, and 0.5 mM stepwise titration of 1 mM carbonyl cyanide 4-(trifluoromethoxy)phenylhydrazone (FCCP) added until maximal uncoupling (uncoupled state). In experiment 2 (octanoylcarnitine-driven respiration), rates were measured with 200 mM octanoylcarnitine + 1 mM malate, 2 mM ADP, 2 mM glutamate + succinate, 4 mg/mL oligomycin, and 1 mM stepwise titration of FCCP. Cytochrome c (10 mM) was used to determine mitochondrial membrane integrity.

### 2.7. Western Blot Analysis

Aorta tissue (control group *n* = 5, sepiapterin *n* = 4) was ground under liquid nitrogen in mammalian cell lysis buffer (M-PER with 150 mM NaCl, 1 mM EDTA, 1 mM EGTA, 5 mM Na_4_P_2_O_7_·10H_2_O, 1 mM Na_3_VO_4_, 20 mM NaF, 500 mM okadaic acid, and 1% protease inhibitor cocktail). Protein concentrations were determined using the Bradford assay, and 15 *μ*g to 40 *μ*g protein were resolved on either 10% or 12% SDS-PAGE gel. Proteins were detected by florescence instrumentation from LI-COR Biosciences, and densitometry was performed in the software Image Studio (version 4.0). All data is normalized to *β*-actin protein expression, and specific activity was calculated as phosphorylated protein expression normalized to total protein expression.

### 2.8. Statistics

Two-tailed Student *t*-test, Holm-Sidak method for multiple comparisons, or repeated-measures two-way ANOVA were used for data analysis. A *p* value of less than 0.05 was considered statistically significance in all tests.

## 3. Results

### 3.1. Impact of Sepiapterin on Metabolic Parameters

Metabolic parameters were assessed at baseline and endpoint of the study. As expected, there were no significant differences in any of the metabolic parameters at baseline (Table 1, *p* > 0.05 for all comparisons). Both groups demonstrated significant weight gain (Table 1, *p* < 0.001 for time effect), but were not different from each other (Table 1). At the end of the study, morning ad libitum fed glucose concentrations were significantly decreased in both groups as compared with baseline concentrations (Table 1, *p* < 0.02 for time effect), and ad libitum insulin concentrations were significantly elevated as compared with baseline concentrations in both groups (Table 1, *p* < 0.005 for time effect, *p* > 0.05 for group effect). At endpoint, we observed a trend for lower ad libitum and fasting glucose concentrations in sepiapterin-treated animals, but this was not statistically significant; both ad libitum and fasting insulin concentrations were similar between groups in both states (Table 1). No differences in endpoint triglyceride concentration were observed (Table 1).

### 3.2. Sepiapterin Improves Insulin-Mediated Glucose Tolerance in Sedentary Animals

**Figure 1:**
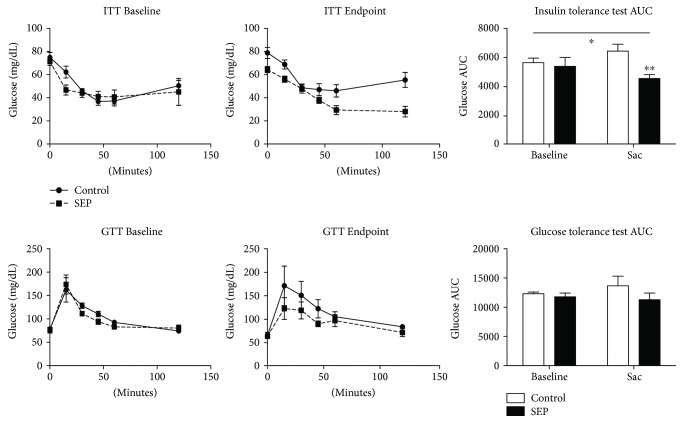
Insulin and glucose intraperitoneal tolerance test. Animal insulin and glucose tolerance were assessed at baseline and end of the study (sac). Control group *n* = 5, SEP *n* = 4 at baseline, control group *n* = 5, and SEP *n* = 3 at endpoint. ^∗^Interaction effect of *p* < 0.05; ^∗∗^difference at sacrifice as determined by post hoc analysis (*p* < 0.05) and repeated-measures two-way ANOVA.

Insulin (IP-ITT) and glucose tolerance tests (IP-GTT) were conducted at baseline and endpoint. The area under the curve (AUC) of glucose concentrations of both control and sepiapterin groups was analyzed. At the end of the study, a significant interaction effect of time and treatment was observed when comparing ITT AUC of animals treated with sepiapterin and the control animals across the study (Figure 1, *p* < 0.03). According to a post hoc analysis, sepiapterin-treated animals had a significantly less AUC during the ITT at sac as compared with control animals (*p* < 0.03). GTT AUC were not significantly different across the study or between groups (Figure 1).

### 3.3. Sepiapterin-Mediated Improvements in Vasodilation

**Figure 2:**
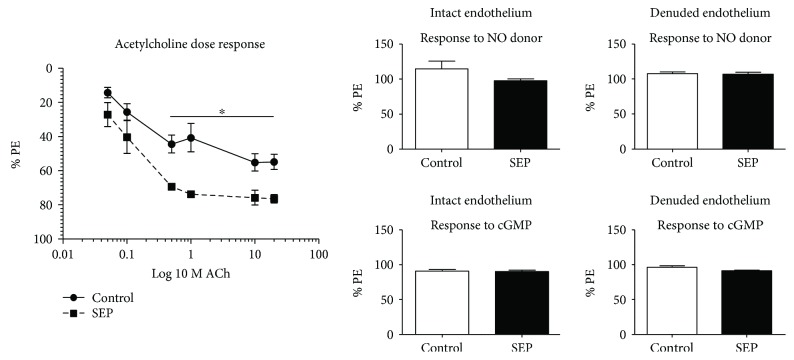
(a, b, and c) Aorta vasodilation. At the end of the study, aorta rings (2 mm) were hung in an upright water muscle bath and analyzed for vasodilation using a dose response to ACh (a) and NO donor S-nitroso-N-acetyl-DL-penicillamine (SNAP, b) and cGMP (c) in intact and denuded endothelium. Control group *n* = 5 and SEP *n* = 4. Data are presented as mean ± SEM, ^∗^*p* < 0.05, Student's *t*-test.

Animals' aortae were analyzed for vasodilation and vasoconstriction using a muscle bath apparatus. Aorta of sepiapterin-treated animals exposed to a dose response curve of ACh, a stimulator of vasodilatation via NOS activation, showed significantly higher responses at concentrations 0.5, 1, 10, and 20 *μ*M, as compared with control animals (Figure 2(a), *p* = 0.01, 0.03, 0.03, and 0.01, resp.). When aortae were treated with a NO donor, SNAP, no differences were observed between control and treated tissue either intact or denuded (Figure 2(b), *p* > 0.05). No differences were seen between groups with cGMP (Figure 2(c), *p* > 0.05).

### 3.4. Sepiapterin Had No Effect on Vasoconstriction

**Figure 3:**
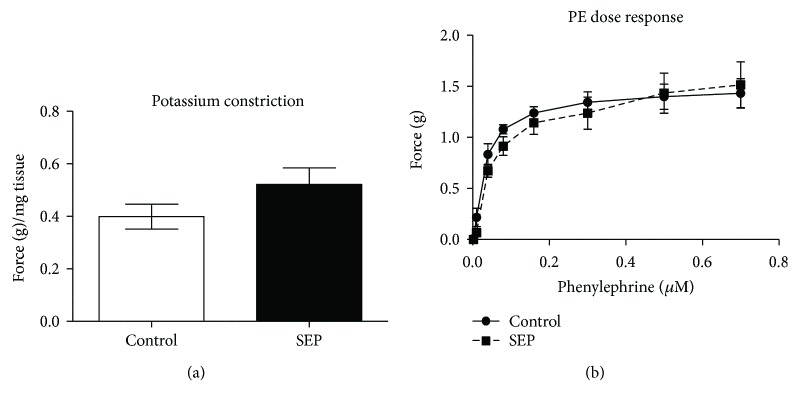
(a and b) Aorta vasoconstriction. At the end of the study, aorta rings (2 mm) were hung in an upright water muscle bath and analyzed for vasoconstriction using high potassium buffer (a) and a dose response to phenylephrine (PE, b). Control group *n* = 5 and SEP *n* = 4. Data are presented as mean ± SEM, Student's *t*-test.

Aorta from sepiapterin-treated animals exposed to high potassium buffer, designed to create a depolarizing contractile effect, showed no difference in contraction as compared with control tissue (Figure 3(a)). Following a dose response curve of phenylephrine, there were no differences in vasoconstriction between groups (Figure 3(b)).

### 3.5. No Differences Noted in Aorta Respiration during PMG-Driven Respiration

**Figure 4:**
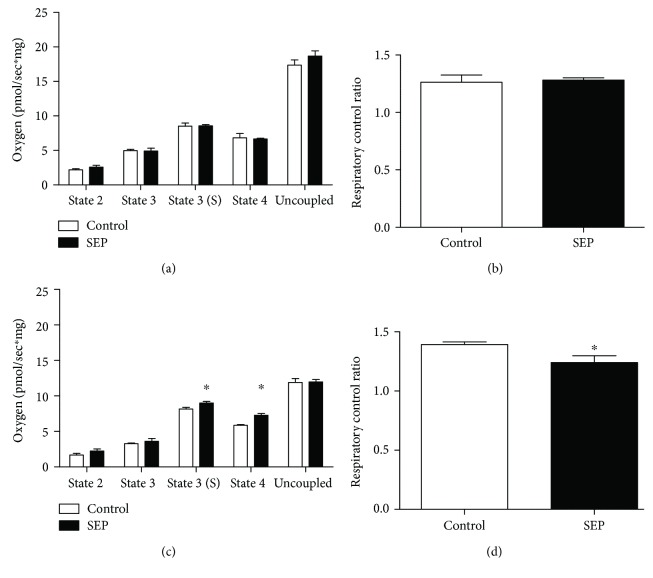
(a–d). Mitochondrial respiration. Aorta from animals were permeabilized and exposed to two different experiments using various substrates (a, b: pyruvate, malate, glutamate, ADP, cytochrome c, succinate, oligomycin, and carbonyl cyanide-4-(trifluoromethoxy)phenylhydrazone (FCCP)) or (c, d: octanoylcarnitine, malate, glutamate, ADP, cytochrome c, succinate (S), oligomycin, and (FCCP)). State 2 respiration (S2), state 3 respiration (S3), and state 4 respiration (S4) were measured. States were analyzed as shown above, including respiratory control ratio (RCR, state 3(S)/state 4). Control group *n* = 5 and SEP *n* = 4. Data are presented as mean ± SEM, ^∗^*p* < 0.05, Student's *t*-test.

Mitochondrial function is required for optimal vasoreactivity, so we next examined aortic mitochondrial oxygen consumption in the context of two different substrate-uncoupler-inhibitor titrations (SUIT) representing carbohydrate- (PMG-) and lipid- (octanoylcarnitine-) driven respiration. Aorta from animals were harvested, permeabilized with saponin, and exposed to substrates and inhibitors mimicking the tricarboxylic acid (TCA) cycle and modulating mitochondrial function. No differences were observed in any respiration states or RCR of PMG-driven experiments (Figures 4(a) and 4(b), *p* > 0.05).

### 3.6. Aorta from Sepiapterin-Treated Animals Show Increased Respiration and Decreased Respiratory Efficiency during Octanoylcarnitine-Driven Respiration

During octanoylcarnitine-driven mitochondrial respiration, state 3 with succinate (state 3S) and state 4 were significantly higher in aorta from animals treated with sepiapterin (*p* = 0.04 and <0.001, resp., Figure 4(c)). RCR was significantly lower in octanoylcarnitine-driven respiration in those treated with sepiapterin as compared with control animals (*p* = 0.03, Figure 4(d)).

### 3.7. Cellular Signaling Regulating Mitochondrial Function Is Not Impacted by Sepiapterin Treatment

**Figure 5:**
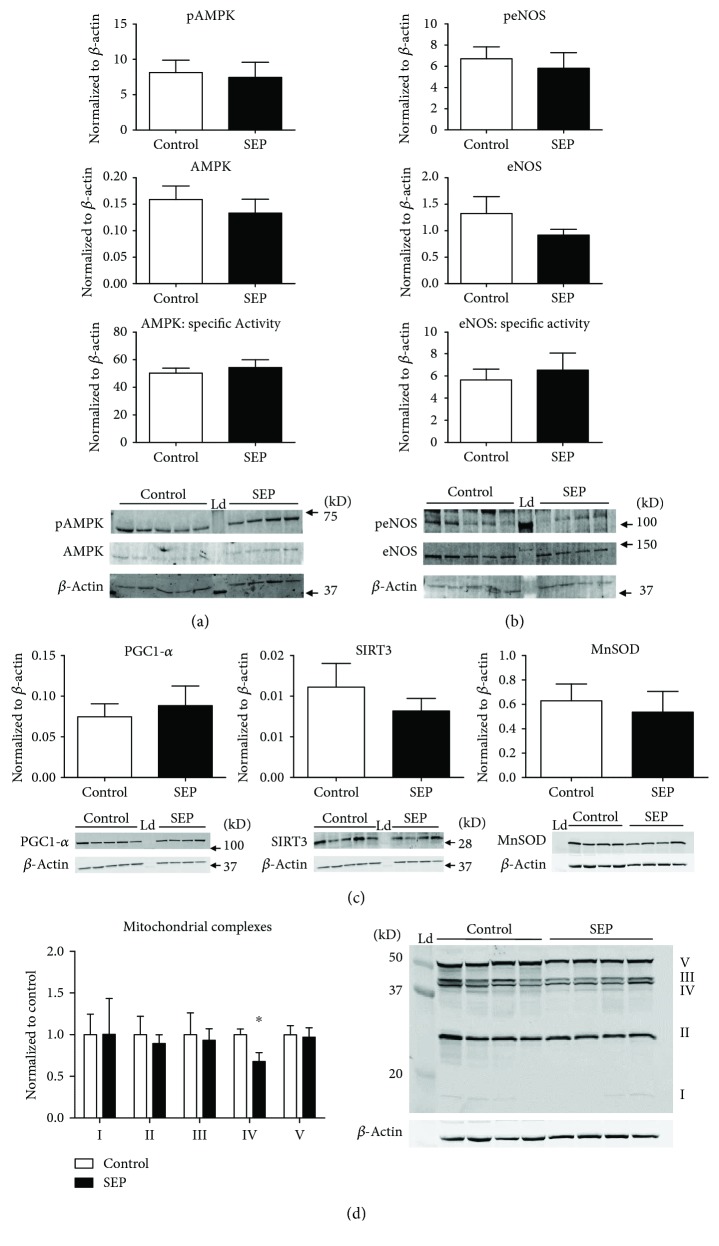
(a–d) Cellular signaling in aorta tissue: aorta tissue was harvested and lysates processed for Western blot analysis. Blots of aorta were probed for pAMPK, AMPK (a), peNOS, eNOS (b), PGC-1*α*, SIRT3, MnSOD (c), and mitochondrial complexes (d). In (d), complex I was calculated using enhanced blot image for visibility and was found not to be significantly different from control (data not shown on blot image). Ladder (Ld) was loaded to orient protein identification, and *β*-actin was used for normalization. Specific activity was calculated as phosphorylated protein expression normalized to total protein expression. Control group *n* = 5 and SEP *n* = 4. Data are presented as mean ± SEM, ^∗^*p* < 0.05, Student's *t*-test.

Lysates of aortic tissue from animals were analyzed for protein expression using Western blot. There were no differences in expression of AMPK or NOS-specific activity or in PGC-1*α* expression between the groups (Figures 5(a) and 5(b), *p* > 0.05 for all).

### 3.8. Decrease in Mitochondrial Complex IV Expression in Aorta from Sepiapterin-Treated Animals Was Observed

Protein expression of mitochondrial complex IV in rat aorta was significantly decreased (Figure 5(d), *p* < 0.05). There were no other changes noted in complex expression (Figure 5(d)). Protein expression of MnSOD, a mitochondrial antioxidant, was unchanged in sepiapterin-treated rats as compared with controls (Figure 5(c)).

## 4. Discussion

Sedentary behavior is increasing worldwide and correlates with elevated risk of abnormal metabolism, cardiovascular disease, and cancer [1–3, 9]. In cohort studies and preclinical studies, sedentarism is associated with endothelial dysfunction [4–8]. Endothelial dysfunction is caused by abnormalities in vascular function at the cellular level and heralds the progression of pathology, leading to hypertension and other cardiovascular disease [33]. Endothelial NOS is crucial for endothelial function and sensitive to factors such as insulin resistance and changes in glucose and free fatty acids related to sedentarism [7, 11]. In general, supporting physiological homeostasis is an effective strategy to increase resilience in a cell-specific manner. Here, we tested the hypothesis that sepiapterin, an oxidation-resistant precursor of BH_4_, would support eNOS function and preserve vasoreactivity in sedentary animals. Treatment with sepiapterin improved ACh-mediated vasodilatation, suggesting endothelial dysfunction in the untreated rats. Interestingly, sepiapterin-treated rats had decreased ad libitum and fasting glucose concentrations and increased insulin sensitivity without a change in glucose tolerance, indicating a potentiation of insulin action in the presence of sepiapterin. Taken together, these results suggest that sedentarism contributes to changes in vascular function and metabolism that are responsive to sepiapterin.

Supporting cellular eNOS function by restoration of a cofactor commonly depleted in dysmetabolism effectively elevated vasodilation. Our laboratory has reported that NOS activity is compromised in an insulin-resistant rat model, resulting in vascular abnormalities [11]. We have also shown that NOS function and mitochondrial adaptation are interdependent; both were impaired in vascular cells isolated from a diabetes and control rat model exposed to glucose challenge, illustrating the cellular perturbation downstream of NOS in a context of metabolic abnormality [11, 16]. We also reported the beneficial effects of targeting eNOS/cAMP activity using a dipeptidyl peptidase inhibitor 4 (DPP-4, saxagliptin) on mitochondrial function, vasoreactivity, and the adaptive exercise training response [34]. In the current study, we tested the impact of targeting eNOS activity with sepiapterin and demonstrated improved vasodilation, perhaps by increasing BH_4_ concentrations, an essential cofactor for eNOS activity. The strength of this strategy is that this cofactor alone does not change vascular tone; it specifically impacts receptor-mediated vasodilation. These findings are consistent with previous observations indicating that sedentary behavior causes endothelial dysfunction responsive to intervention [14, 35].

Our results confirm that sepiapterin supplementation improves endothelium-dependent vasodilation in sedentary rats, as previously described [25–27]. Sepiapterin is a compound uniquely suited to supporting NOS activity in the context of metabolic distress due to its stability in a climate of excess ROS. We did not show any significant differences between groups when tissues from both were exposed to a NO donor of cGMP, suggesting that the impact of sepiapterin is on the endothelium. Sepiapterin increases the NOS cofactor BH_4_, a support of eNOS enzyme activity, and does not specifically target eNOS; thus, we may not expect to see impacts on eNOS protein expression. We conclude that sepiapterin specifically affects endothelial-dependent vasodilation in our aortae.

Mitochondria are necessary for vasomotion [36]; we have reported abnormal mitochondrial content, function, and adaptation to stress (nutrient or exercise) in the context of metabolic stress and NOS deletion or inhibition [11, 16]. We hypothesized that supporting NOS activity with sepiapterin would affect mitochondrial function. Our new data show that sepiapterin increases aortic mitochondrial oxygen consumption during octanoylcarnitine-driven respiration. This finding agrees with recent literature showing a switch to mitochondrial usage of lipid fuel during physiologically stressful or demanding conditions [37]. Sepiapterin stimulation of mitochondrial activity could be either in a NOS-dependent or independent mechanism, resulting in a need for differential fuel utilization to accommodate greater rates of respiration. In turn, the observed effect on vasodilation may have required an increase in ATP production, thus resulting in greater respiration activity. We and others have shown that NOS regulates mitochondrial function, in part, due to NO and NOS activation [15–18]. Thus, with NOS activity supported, these novel observations are biologically plausible. We did not observe a significant change in the mitochondrial biogenesis signaling proteins, AMPK/PGC-1*α*, SIRT3, or mitochondrial proteins in this sedentary rat model, with the exception of complex IV. Lack of difference in these regulators of mitochondrial biogenesis and function may be due to temporal completion of mitochondrial adaptation and resolution of acute signaling, despite the observed chronic impacts on mitochondrial respiration. Also, impact of sepiapterin treatment on cellular signaling might be subtle and require a larger sample size to detect. Alternately, differences in respiration rates may be independent of complex expression and related to fuel partitioning.

We report higher mitochondrial oxygen consumption with a lipid substrate in those animals treated with sepiapterin. Specifically, our experiments show a significantly greater respiration rate in state 3 and state 4 of aorta from sepiapterin-treated animals at the end of the study, but no differences in PMG-driven respiration rates. Further analysis reveals a significantly decreased RCR in animals treated with sepiapterin. RCR is an indicator of respiration efficiency. The physiological implications of changing aortic fuel preference are unclear. In a recent report, the peroxisome proliferator-activated receptor delta (PPAR*δ*) ligand had a similar impact in skeletal muscle to selectively improve fatty acid oxidation [37]. These authors interpreted this change in substrate preference as a glucose sparing effect which, in their hands, was related to improved exercise endurance [37]. While the molecular targets of sepiapterin and PPAR*γ* differ, both interventions demonstrate enhanced fuel partitioning to lipid that is mediated by targeting intracellular machinery (eNOS or PPAR*δ*). Each improves either vascular or skeletal muscle health, respectively. We speculate that fuel switching in the vasculature may be related to improved vasoreactivity. The differential rate of mitochondrial respiration between the two sets of substrates is intriguing and suggests that NO may impact mitochondrial fuel utilization, or substrate flux, and thus respiration in the presence of certain compounds integral to the TCA cycle.

Sedentary behavior is believed to deleteriously impact insulin action in addition to its impact on the vasculature. We therefore characterized the metabolic status of our rats. We observe that sepiapterin also decreases ad libitum and fasting glucose concentrations and improves insulin sensitivity and improves peripheral insulin action. These data agree with previous studies showing an intimate link between NOS and insulin action [38–40]. One study describes an insulin-mimetic activity of NOS; shown to be stimulated by insulin, NO acted in an insulin-like manner, resulting in improved glucose uptake [38]. Taken together, these findings indicate a multifactorial impact of sepiapterin on endothelial-dependent vasodilation and insulin action.

This study has some limitations. Sepiapterin is a known precursor of BH_4_; still this pharmacological compound could have mechanisms of action beyond eNOS that contribute to the results reported. We do not include direct measurements of BH_4_, largely because of the ongoing cyclic metabolism of BH_4_ to BH_2_. Any concentrations may only reflect the cycle at the time of plasma collection. Also, we only have two experimental groups; a future study will include an exercised animal group. Our sample size was based on an a priori power calculation, but it is small: however, with this sample size, we observed significant effects of sepiapterin treatment on vasodilatation and mitochondrial function, our primary endpoints. Although we verify sepiapterin food consumption by the animals, the exact dosage is unknown. We did not measure ROS or proxy indicators of ROS. We also do not have direct measurements of NO content as a result of sepiapterin supplementation. NO has a short half-life, making it difficult to measure. Future experiments in our laboratory will determine NO as a result of chronic interventions. Also, we make no assumption that there is a connection with the vasomotion improvement in the aorta (conduit artery) and improved systemic insulin action. Rather, we show that even in large conduit vessels, we observe physiological changes with treatment at both tissue and cellular levels. This is an important observation and may indicate a comprehensive and multitargeted response to interventions at several sites in the vasculature. Regardless of these limitations, we verify known mechanisms of NO signaling, particularly in insulin sensitivity and vasodilation, consistent with increased NOS activity.

## 5. Conclusion

Our work herein supports the successful targeting of NOS via sepiapterin supplementation resulting in improved vasodilation and enhanced insulin sensitivity. This work highlights that sedentarism decreases both vascular function and insulin sensitivity. These improved functional endpoints, and their regulation at the cellular level, may or may not be interdependent. We ultimately conclude that NOS is critical for vascular function, as is well known, and likely also important for glucose regulation, specifically insulin-mediated glucose disposal. Therapeutics that target the activity of NOS may be important for alleviating metabolic disruptions associated with sedentary behavior. In summary, novel agents to restore the deleterious impact of sedentary behavior on NOS function should be pursued for both their cardiovascular and metabolic benefits [2, 3, 9].

## Figures and Tables

**Table 1:** Metabolic parameters. Animal glucose (control *n* = 5, sepiapterin (SEP) *n* = 4) and insulin (control *n* = 3, SEP *n* = 4) concentrations were measured in animals with food ad libitum and following a 6-hour fast. Data are mean ± SEM with ^∗^*p* < 0.05 significant effect of time, repeated-measures two-way ANOVA. ^∗∗∗^Undetectable < assay limit of 0.02 ng/mL.

Group	Weight (g)	Triglycerides (mg/dL)	Glucose ad libitum (mg/dL)	Glucose fasting (mg/dL)	Insulin ad libitum (ng/mL)	Insulin fasting (ng/mL)
Baseline						
Control	340 ± 14	—	77 ± 8	75 ± 4	0.130 ± 0.02	^∗∗∗^
SEP	359 ± 12	—	74 ± 2	71 ± 2	0.332 ± 0.07	0.110 ± 0.05

Endpoint						
Control	544 ± 22^∗^	84.300 ± 25.12	63 ± 2^∗^	79 ± 5	3.260 ± 0.73^∗^	0.468 ± 0.09
SEP	557 ± 23^∗^	88.350 ± 29.95	58 ± 3^∗^	66 ± 2	3.835 ± 0.58^∗^	0.471 ± 0.1

## Data Availability

The data used to support the findings of this study are available from the corresponding author upon request.
